# Web-Based Delivery of a Family-Based Dating Violence Prevention Program for Youth Who Have Been Exposed to Intimate Partner Violence: Protocol for an Acceptability and Feasibility Study

**DOI:** 10.2196/35487

**Published:** 2022-08-05

**Authors:** H Luz McNaughton Reyes, Eliana Gabriela Armora Langoni, Laurel Sharpless, Natalie Blackburn, Agnieszka McCort, Rebecca J Macy, Kathryn E Moracco, Vangie A Foshee

**Affiliations:** 1 Department of Health Behavior Gillings School of Global Public Health University of North Carolina at Chapel Hill Chapel Hill, NC United States; 2 Injury Prevention Research Center University of North Carolina at Chapel Hill Chapel Hill, NC United States; 3 School of Social Work University of North Carolina at Chapel Hill Chapel Hill, NC United States

**Keywords:** dating violence, adolescents, family-based prevention, web-based delivery, feasibility and acceptability, mobile phone

## Abstract

**Background:**

Children exposed to intimate partner violence (IPV) between caregivers are at an increased risk of becoming involved in dating violence during adolescence. However, to date, few adolescent dating violence (ADV) prevention programs have been developed for and evaluated with youth exposed to IPV. An exception is Moms and Teens for Safe Dates (MTSD), an evidence-based ADV prevention program for mothers or maternal caregivers (mothers) exposed to IPV and their teenagers. The MTSD program comprises a series of booklets that families complete together in a home that includes activities to promote positive family communication and healthy teenager relationships. We developed a web-adapted version of the MTSD program—entitled *eMoms and Teens for Safe Dates* (eMTSD)—to provide a delivery format that may increase program appeal for digitally oriented teenagers, lower dissemination costs, lower reading burden for low-literacy participants, and incorporate built-in cues and reminders to boost program adherence.

**Objective:**

This protocol is for a research study that has the following three main objectives: to assess the acceptability of eMTSD; to identify the feasibility of the research process, including program adherence and participant recruitment and assessment; and to explore the acceptability, feasibility, and preliminary efficacy of 2 features—text reminders and the creation of an *action plan* for engaging with the program—that may increase program uptake and completion.

**Methods:**

Approximately 100 mothers and their teenagers will be invited to complete eMTSD, which includes six 30-minute web-based modules over a 6-week period. Mothers will be recruited through community organizations and social media advertising and will be eligible to participate if they have at least 1 teenager aged 12 to 16 years living with them, have experienced IPV after the teenager was born, are not currently living with an abusive partner, and have access to an internet-enabled device. Using a factorial design, enrolled dyads will be randomized to the following four *adherence support* groups (n=25 dyads per group): text reminders and action planning, text reminders only, action planning only, and no adherence supports. All participants will complete brief web-based assessments at enrollment after each module is completed, after the full program is completed, and 90 days after enrollment. Program adherence will be tracked using website use metrics.

**Results:**

The data collected will be synthesized to assess the acceptability of the program and the feasibility of the study procedures. An exploratory analysis will examine the impact of adherence support on program completion levels. In November 2021, ethical approval was received and recruitment was initiated. Data collection is expected to continue until December 2022.

**Conclusions:**

The web-based delivery of a family-based healthy relationship program for teenagers exposed to IPV may offer a convenient, low-cost, and engaging approach to preventing ADV. The findings from this study are expected to guide future research.

**International Registered Report Identifier (IRRID):**

DERR1-10.2196/35487

## Introduction

### Background

Each year, approximately 15 million children in the United States are exposed to intimate partner violence (IPV) between their parents or other caregivers [1], with >25% of children being exposed to IPV in their lifetime [2]. Research suggests that IPV exposure, broadly defined as direct witnessing, hearing, or seeing the aftermath of any form (eg, physical or psychological) of violence between caregivers [3], is a multifaceted traumatic experience that can have adverse impacts on children’s cognitive (eg, biases in information processing), emotional (eg, anger dysregulation), and social development (eg, deviant peer affiliation) [4]. Children who experience these adverse impacts, in turn, are at increased risk of involvement in abusive dating and intimate partnerships during adolescence and adulthood [5,6], a pattern referred to as the *intergenerational* transmission of IPV [7].

The intergenerational transmission of IPV may be disrupted by programs that effectively work to interrupt the processes leading to adolescent dating violence (ADV) among youths exposed to IPV. However, although numerous studies have identified programs that prevent dating violence among general samples of youth, relatively little research has been conducted to develop or evaluate dating abuse prevention programs among high-risk youth, such as those who have been exposed to interparental IPV [8]. Furthermore, despite research suggesting that parents and other caregivers play a key role in adolescent relationship development, scant research has been conducted to develop and evaluate family-based approaches to ADV prevention [9]. An exception is Moms and Teens for Safe Dates (MTSD), a family-based dating abuse prevention program developed for youth who have been exposed to IPV and their mothers or maternal caregivers (hereto forth referred to as *mothers* and inclusive of nonbinary people and gender expansive and transgender women who identify as mothers or maternal caregivers) who experienced the abuse [10]. The MTSD program is designed to promote a family environment that is protective against dating abuse and comprises a set of 6 printed booklets with interactive activities that mothers and teenagers complete together (self-administer) in the home. In a randomized controlled trial, the program was found to be effective in increasing family cohesion and preventing dating abuse among youth with higher but not lower levels of IPV exposure [11].

Given that the MTSD program was found to have positive impacts, the next step in the continuum of research on the program is to optimize the program for dissemination (ie, distribution of the program by the community and other agencies to mothers exposed to IPV and their teenagers) and implementation (ie, use of the program by mothers and their teenagers within the real-world family setting). Notably, the MTSD program was structured to avoid 2 main obstacles in the implementation of family-based interventions. First, it avoids logistical barriers that prevent families from attending programs offered at out-of-home locations as the program can be conducted at home (or at any location the family chooses). Second, there are no costs associated with training or paying the delivery staff as the program is self-administered. However, the drawbacks of the program from a dissemination and implementation (D&I) standpoint include the costs of printing and mailing (eg, through the postal service) the booklets, which may be prohibitive to organizations serving mothers exposed to IPV, which are typically low-resourced community-based organizations [12]; insufficient reach to low-literacy members of the target population, given the relatively high reading burden of the program; and a lack of built-in prompts or cues to engage with the program, which may result in poor user engagement in real-world settings. For example, in the MTSD randomized controlled trial, which provided small financial incentives for booklet completion, 62% of families reported completing the final booklet [11].

The research team proposed that each of these D&I barriers could be addressed by adapting the program for web-based delivery. In particular, web-based delivery has the potential to reduce dissemination costs; allow for the audiovisual presentation of information and activities, thus potentially reducing the reading burden and improving understanding among audiences with lower health literacy [13,14]; and allow for automated delivery of reminders, tailored based on website use, to maximize program uptake and completion. Furthermore, the research team postulated that a digital platform for MTSD may increase program appeal among teenager participants, given research suggesting that there has been a pronounced shift among US teenagers away from the use of *legacy* media (eg, books) and toward digital media [15].

### Adapting the MTSD Program for Web-Based Delivery

To develop a web-adapted version of the MTSD program, the research team followed a step-by-step process guided by the Integrate, Design, Assess, and Share framework for developing effective digital interventions [16]. Briefly, stages of the adaptation process included revision of the conceptual model, ideation of potential web-based representations of booklet material, iterative prototype development conducted in collaboration with a mother-teenager advisory board, 3 cycles of prototype user testing and refinement conducted with 6 mothers exposed to IPV and their teenagers, and a *soft launch* of the complete program and study procedures with a different set mother-teenager dyads (n=8) exposed to IPV. For the soft launch, mothers were provided with a link to the web-based program and asked to complete it with their teenagers over a 3-week implementation period. Feedback on design and functionality was obtained through surveys embedded in the website, a follow-up web-based survey, and a brief telephone exit interview. Informed by usability research with low-literacy parents, we prioritized the simplicity of design, content, and technical features during the first phases of prototype development [17]. As part of this process, several text-based program activities were converted to multimedia instructions—communications that use words in combination with graphics, animated videos, and other audiovisual experiences, which have been shown to improve understanding among low-literacy parents compared with the presentation of text alone [18]. Mother and teenager feedback on the program was incorporated across all development phases, consistent with user-centered design principles [19]. Specifically, feedback from mothers and teenagers was used to make decisions about the look and feel of the website; update the program to reflect current adolescent dating language and practices; represent a diverse set of mothers, teenagers, and relationships; and ensure that activities presenting teenager dating and family communication scenarios were realistic. The finalized program, which is titled *eMoms and Teens for Safe Dates* (eMTSD), comprises 6 30-minute modules that can be completed on any device with access to the internet (eg, smartphones, tablets, and computers).

### Objectives

Here, we present a protocol for a feasibility and acceptability study of eMTSD, which is a necessary step in the continuum of research on this program. This study has the following three main objectives: (1) to assess the acceptability of the eMTSD program; (2) to identify the feasibility of the research process, including program adherence and participant recruitment and assessment; and (3) to explore the acceptability, feasibility, and preliminary efficacy of 2 *adherence support* factors—SMS text message reminders and action planning—that may increase eMTSD program uptake and completion.

## Methods

### Study Design Overview

**Figure 1:**
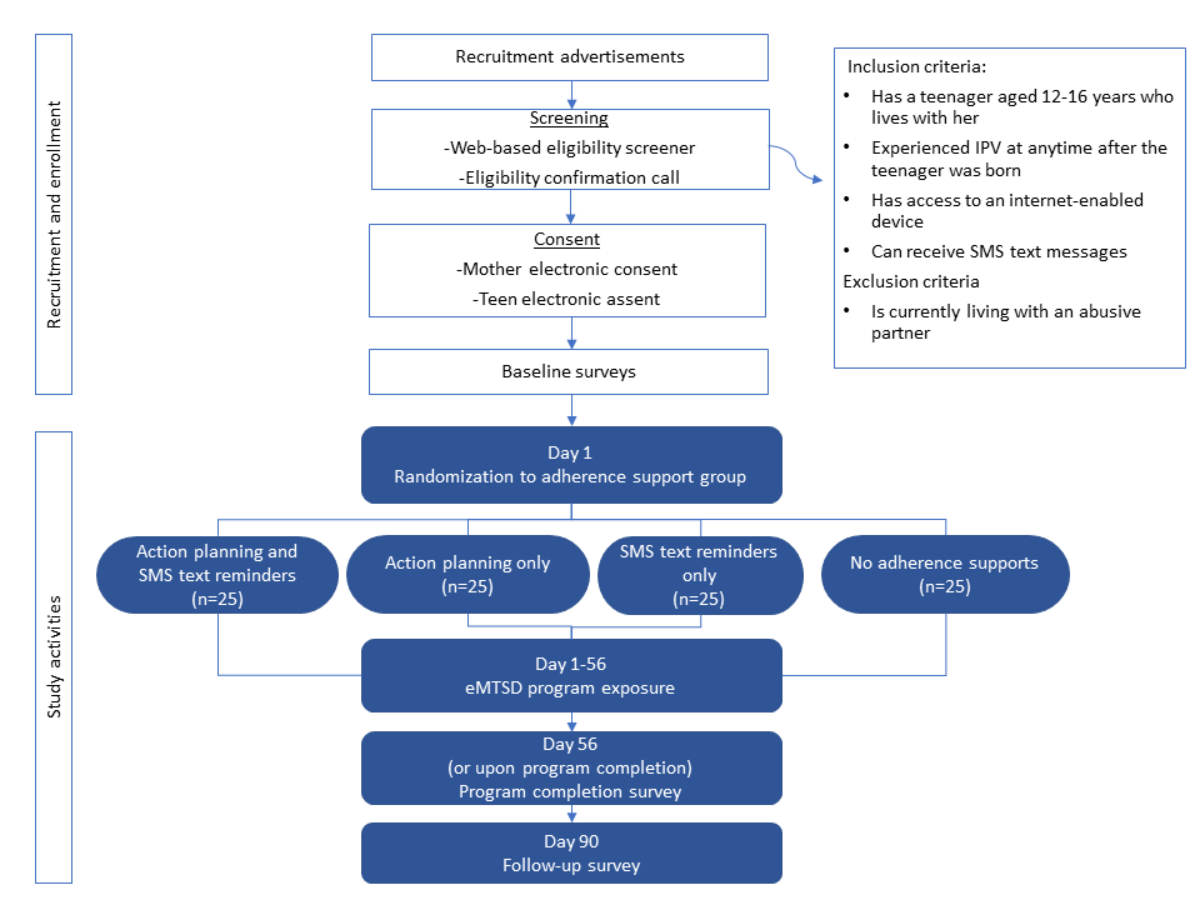
Study flow. eMTSD: eMoms and Teens for Safe Dates; IPV: intimate partner violence.

The flow of this study is summarized in Figure 1. Approximately 100 mother-teenager dyads, who may reside anywhere in the United States, will be enrolled in the study. Eligible mothers will have a teenager aged 12 to 16 years living in the home, have experienced IPV at some point after their teenager was born, and not be currently living with an abusive partner. Mother-teenager dyads who enroll will be randomized to 1 of 4 *adherence support* groups: text reminders only, action planning only, text reminders plus action planning, or no adherence support. Mothers in the text reminder groups will receive weekly SMS text messages tailored to their program completion level, which will cue them to engage with the program. Mothers in the action planning groups will complete a form before initiating the program, which will ask them to think about when and where they will work on the program with their teenagers. SMS text message reminders and action planning are referred to as *adherence support factors* as they are posited to increase the uptake and completion of program activities by mothers.

All enrolled mother-teenager dyads, regardless of adherence support group assignment, will be asked to complete the eMTSD program together over a 6-week period with a 2-week grace period allowed for families who are unable to comply with this program completion schedule (ie, up to a maximum of 8 weeks will be allowed to complete the program). Feasibility and acceptability outcomes, described in more detail in the following sections, will be assessed in several ways. First, a study tracking database will allow us to calculate recruitment, enrollment, and retention rates. Second, website use data will allow us to assess program uptake, engagement, and adherence. Third, web-based surveys will be administered to mothers and teenagers at baseline (enrollment), after program completion (maximum of 8 weeks after baseline), and 90 days after baseline (follow-up). These surveys will assess program acceptability and provide information on the feasibility of using web-based surveys to collect data on healthy relationship cognition, skills, and behaviors. Finally, program acceptability will be assessed using brief (1-2 minutes) module acceptability surveys that appear in the web-based program at the end of each module. For each participant, the study is expected to last for approximately 90 days from enrollment to the follow-up assessment.

### Participants

Eligible participants are mothers residing in the United States who (1) have at least 1 child aged 12 to 16 years (hereafter referred to as their *teenager*) who lives with them at least part of the time, (2) have experienced IPV at some point in their lives after at least 1 of their teenagers aged 12 to 16 years was born, (3) are not currently living with an abusive partner, (4) are able to read and speak English, (5) have access to an internet-enabled device, and (6) are able to receive SMS text messages. The age range of 12 to 16 years age range was selected based on evidence indicating that this is an appropriate developmental period for programs focused on the primary prevention of ADV [20].

### Recruitment

We aim to recruit approximately 120 eligible mother-teenager dyads and enroll 100 dyads (80% of which will be eligible) over an 8-month period (approximately 13 dyads enrolled per month). Recruitment, which will target potential mother participants, will occur through study advertising via Facebook or Instagram, Craigslist, and Reddit posts and via information dissemination through community agencies (eg, social services organizations) and educational institutions (eg, community colleges) that work with or provide services to mothers, survivors of IPV, and youth exposed to IPV. Study advertisements will direct individuals who are interested in participating to an initial web-based eligibility screener. If the participants want more information, the study telephone number and email address will be provided. Advertisements will also list the study website, which includes detailed information about the study for potential participants and the link to the initial eligibility screener.

### Enrollment and Randomization

Potentially eligible participants who contact the study, either by completing the web-based screener or by calling the study phone number, will complete a full eligibility screener with staff on the telephone. During this call, the study will be described in detail, and staff will gauge their interest in participating. Mothers with >1 age-eligible teenager will be further asked to select one teenager to participate in the study or, if they prefer, to allow study staff to randomly select the teenager who will participate. Mothers who indicate that they are interested in enrolling will be emailed links to a web-based consent form and parental permission form for them to complete, as well as an assent form for their teenager to complete. Once the consent and assent forms are completed, the study team will send the mother participant links to the baseline surveys via 2 emails (one email with the link for the mother baseline survey and another with the link to the teenager baseline survey). Mother-teenager dyads who complete the consent, assent forms and baseline surveys, all of which will be completed on the web, will be considered enrolled in the study and randomized to 1 of the following 4 *adherence support* groups: (1) action planning only, (2) SMS text message reminders only, (3) action planning and SMS text message reminders, or (4) no support. A computer random number generator will be used to select random permuted blocks with a block size of 8 and an equal allocation ratio. The random assignment table will be generated by the study principal investigator before the initiation of study enrollment. As participants are enrolled and records are added to the system by the study staff, dyads will be assigned a study ID, and the random assignment table will be consulted to assign the dyad to 1 of the 4 adherence support factor groups.

### The eMTSD Program: Program Structure and Conceptual Model

**Figure 2:**
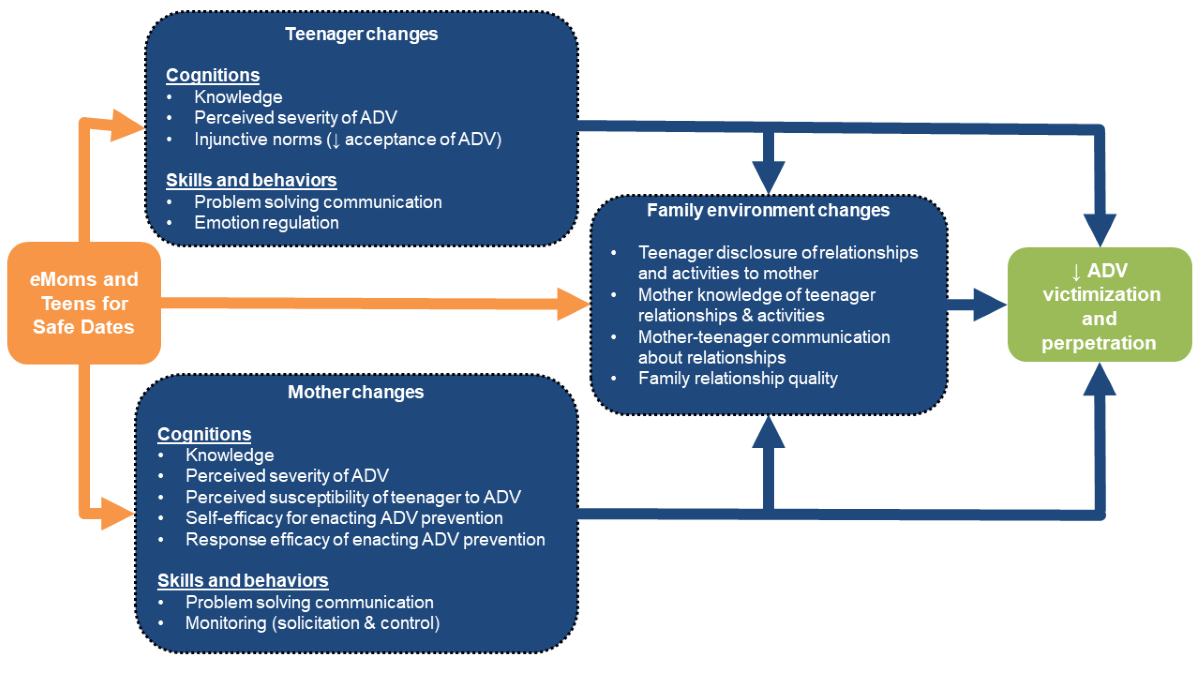
eMoms and Teens for Safe Dates conceptual model. ADV: adolescent dating violence.

The eMTSD program includes 6 modules, each designed to take approximately 30 minutes to complete, which delivers content adapted from the original MTSD program via text, videos, and interactive activities with questions for discussion between the mother and the teenager. Consistent with the original program, the modules aim to increase parent-teenager communication about healthy and unhealthy relationships and reduce teenagers’ risk of experiencing and perpetrating dating abuse. Figure 2 provides a conceptual model, elaborated from the original MTSD conceptual model, which details the specific constructs that the program targets for changes within mothers, teenagers, and at the level of the family dyad, which, in turn, are posited to affect the teenager’s risk of becoming involved in dating violence. As noted by Foshee et al [10], protection motivation theory, cognitive developmental theories, and empirical research have been used to select and specify constructs that MTSD aims to change at the family and individual levels.

**Figure 3:**
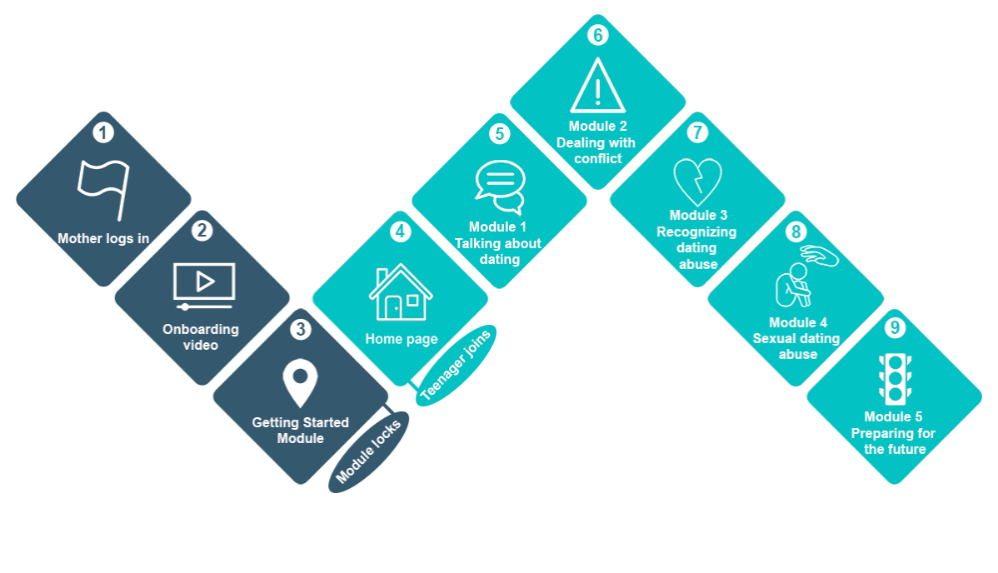
eMTSD program flow. eMTSD: eMoms and Teens for Safe Dates.

Consistent with the format of the original program, the web-adapted program is designed such that the mothers and teenagers must complete the modules in order. Flow through the program, summarized in Figure 3, begins with a *mother-only* phase, during which the mother prepares to engage in the program with their teenager, and then moves into a *mother-teenager* phase, during which the mother and the teenager engage in the program together. Mothers who log into the program are directed to watch a short animated *onboarding* explainer video that describes how the web-based program is organized. Once the onboarding video has been viewed mothers proceed to complete the *Getting Started* module, which is designed to prepare the mothers to engage in the program with their teenagers. At the end of the *Getting Started* module, mothers will be instructed to create a personal identification number for accessing any content in this module in the future and to start the program with their teenagers. To ensure that mothers are given the choice to discuss their IPV history with their teenagers, the *Getting Started* module, which is the only module that includes content addressing the mothers’ IPV history, becomes locked and not viewable to the teenagers after the mothers have completed it. Mothers will then proceed to the program home page, which includes a short video for mothers and teenagers to watch together and then complete 5 modules designed to be completed by mothers and their teenagers together. The five modules for mothers and teenagers to complete together include information and activities focused on (1) positive communication, (2) conflict resolution skills, (3) different forms of dating abuse and the harms they cause, (4) awareness of sexual dating abuse and the importance of consent, and (5) creating family rules and norms around dating relationships. Table 1 summarizes the module goals and sample activities, and Multimedia Appendix 1 provides screenshots of the activities selected from each module.

**Table 1:** Overview of eMoms and Teens for Safe Dates program module goals and sample activities.

Module and goals		Sample activities
**Getting Started (mothers only)**		
	Inform mothers about the program structure	Mothers watch an explainer animated video about the program goals and structure
	Motivate mothers’ engagement with the program	Mothers identify challenges they face in talking to their teenagers about dating and listen to narrated clips about how to overcome them
**Module 1: Talking About Dating**		
	Identify healthy dating goals	Mothers and teenagers identify qualities they would like in a dating partner
	Improve mother-teenager problem-solving communication	Mothers and teenagers identify positive and negative communication skills used in animated microvideos of everyday family conversations
**Module 2: Skills for Handling Conflict**		
	Increase emotion regulation skills	Mothers and teenagers identify events and cues that indicate when they are experiencing an escalation of anger and select strategies they can use for self-calming
	Increase positive conflict resolution skills	Mothers and teenagers watch an animated video of a disagreement between dating partners and practice using problem-solving communication to resolve the conflict
**Module 3: Recognizing Dating Abuse**		
	Increase awareness of different forms of dating abuse and their negative consequences	Mothers and teenagers learn about tactics that can be used to control and manipulate dating partners and identify the tactics being used in different abuse scenarios
	Foster discussion of healthy relationship characteristics	Mothers and teenagers identify characteristics of “healthy” relationships among their friends and families and in the media and discuss what makes a relationship “healthy”
**Module 4: Sexual Dating Abuse**		
	Increase understanding of the meaning of consent in a dating relationship	Mothers and teenagers watch an animated explainer video that highlights the elements of and importance of consent in a dating relationship
	Identify and counter rape myths	Mothers and teenagers read about and discuss false beliefs about sexual dating abuse that shift blame to the victim
	Identify strategies to reduce the risk of experiencing sexual dating abuse	Mothers and teenagers brainstorm strategies they can use to reduce the risk of experiencing sexual dating abuse
**Module 5: Ready, Set, Prepare!**		
	Identify healthy relationship goals	Teenagers identify ways they want to treat dating partners and ways that they want to be treated
	Increase awareness of signs of dating abuse	Mothers and teenagers identify “red flags” that indicate that someone might be experiencing or perpetrating abusive behavior
	Develop safety plans and dating rules	Mothers and teenagers collaboratively develop a set of family guidelines for dating

### Adherence Support Factors

#### Text Message Reminders

A growing body of research suggests the potential of digital *triggers*, such as SMS text messages for fostering engagement in health interventions [21]. Drawing from this research, we developed a set of SMS text message reminders that are personalized by mother and teenager names and tailored based on the completion level. All mother participants will receive SMS text messages welcoming them to the program on the day they are enrolled in the study. Mother participants assigned to the control and action-planning–only conditions who do not log into the program and complete the *Getting Started* module will receive log-in reminders via text 3 and 7 days after enrollment and a text and email reminder 10 days after enrollment (up to 3 reminders).

Mother participants assigned to receive automated SMS text messages will receive up to 8 tailored SMS text message reminders at fixed intervals. Messages 1, 2, and 3 will be sent 3, 7, and 10 days, respectively, after the initial program log-in email and welcome text are sent. Messages 4 through 8 will be sent every 7 days thereafter until they complete all the program modules or the 6-week recommended program completion period is complete. All dyads will be informed that they should complete a minimum of 1 module per week during the study period. Reminders are thus tailored based on whether the dyad is *on trac*k with respect to this recommended completion rate. Mothers who complete the program on time or ahead of schedule or who have already completed the program will receive a *congratulatory message* about their progress. Mothers who have not logged in or are behind schedule receive messages designed to motivate engagement and emphasize the availability of technical support. Examples of reminders are shown in Textbox 1.

**Example SMS text message reminders.**

**Congratulatory reminder for a mother who is on track**

- “Congratulations on completing the Getting Started module of the Moms and Teens program [mom’s name]. You are a superstar! To stay on track, make a plan to complete Module 1 with [teen’s name] by [goal day]! Module 1 will help you and your teen talk about healthy relationships. Need technical support? We want to help! Text back ‘Y’ or call XXX-XXX-XXXX”

**Motivation reminder for a mother that has not logged in**

- “Dear [mom’s name], we re-sent the login information for the Moms & Teens program to your email. This fun, convenient, and free research-based program has been shown to benefit moms and teens. Make a plan now to login this week to complete the Getting Started module and begin the program with [teen’s name] so that you can get back on track to finish the program by [goal day]. If you need technical support to login to the program text back ‘Y’ or call XXX-XXX-XXXX!”

Once the recommended six week program completion period is complete, all participants who have not finished the program will be notified via email and text and asked to complete the program before the two week grace period ends.

#### Action Planning

Gollwitzer [22] proposed a model of action phases for goal attainment, which suggests that people will be more likely to achieve a goal (such as completing the eMTSD program) if they create an *action* plan in the form of implementation intentions that “spell out the where, when, and how of goal striving in advance.” Research supports this strategy, with a meta-analysis finding that implementation intentions have a moderate to large positive effect on goal attainment [23]. Drawing from this research, mother participants assigned to receive action planning groups will receive a modified version of the email communication with the program log-in information, including additional instructions directing them to complete a brief *action planning* form administered through the REDCap (Research Electronic Data Capture; Vanderbilt University) web application. The action planning form asks participants to (1) make an individual goal for when they would like to finish the program, (2) create a plan for where and when they will complete the program modules with their teenagers, and (3) complete 3 if-then statements to identify the potential barriers to completion that may arise and how they will overcome them. Mothers will be instructed that they can print or save the form to refer to if they desire.

### Definitions and Assessment of Feasibility and Acceptability

#### Feasibility

In this study, *feasibility* will be assessed both in reference to the research processes and to the eMTSD program; that is, feasibility outcomes provide information that enables us to assess the questions of *can this study be done* and *can this program be done*. Specifically, we define feasibility as the extent to which (1) the study is successfully conducted with respect to recruitment, randomization, delivery of adherence support, retention, and assessment, and (2) the eMTSD program can be successfully conducted by mothers and teenagers [24,25]. Textbox 2 summarizes the key feasibility outcomes and their indicators.

**Feasibility outcomes.**

**Recruitment rate**

- Number of dyads recruited per month

**Enrollment rate**

- Percentage of eligible dyads who enroll in the study

**Randomization**

- Percentage of enrolled dyads correctly randomized

**Retention rate**

- Percentage of enrolled dyads who complete the follow-up survey

**Adherence supports**

- Percentage of SMS text messages eligible to be delivered and which are sent
- Percentage of mothers eligible for action planning who complete a plan

**Data collection**

- Percentage of missing data within surveys
- Time taken to complete surveys

**Program uptake and adherence**

- Percentage of participants who log in at least once
- Percentage of participants who start the program with their teenager
- Percentage of modules completed or pages viewed
- Percentage of enrolled participants who complete the program
- Adherence index, comprising the sum of the number of completed modules, number of unique visits to the site, and maximum time between visits [26]
- Time taken to complete program modules
- Participant report of facilitators of and barriers to engaging with the program, including technical problems accessing or using the website

#### Acceptability

In this study, acceptability will be assessed in reference to the eMTSD program, as well as with respect to adherence support factors (SMS text message reminders and action planning). Drawing from the Theoretical Framework of Acceptability (TFA), we define acceptability as the extent to which mothers and teenagers consider eMTSD to be appropriate based on their cognitive and emotional responses to the intervention [27,28]. The TFA was applied to develop quantitative indicators assessing the acceptability of the eMTSD across each of the 7 component domains proposed by the TFA (affective attitude, burden, ethicality, coherence, opportunity costs, perceived effectiveness, and self-efficacy). Table 2 lists the acceptability outcomes, including their linkages to each TFA domain.

**Table 2:** Acceptability outcomes.

Outcome and domain			Indicator or indicators
**Program acceptability**			
	Affective attitude	I enjoyed doing the module or program^a^ The module or program kept my attention^a^	
	Burden	The module or program was easy to do^a^ The module or program was too long^a^	
	Effectiveness	I learned useful information from the module or program^a^ The program will reduce my teen’s chances of experiencing dating abuse^a^	
	Ethicality	The program covered topics that are important to me^a^	
	Cohesiveness	I understood what the program is trying to do^a^	
	Self-efficacy	I understood how to complete the program^a^	
	Opportunity cost	Doing the program was time well spent^a^	
	Overall^b^	Percentage of participants who report agreement with all acceptability indicators	
**Adherence support acceptability**			
	Effectiveness	Percentage of participants who report it was helpful to receive text reminders Percentage of participants who report it was helpful to complete an action plan	
	Burden	Percentage of participants who report they received too many SMS text messages	

^a^Program acceptability indicators will be operationalized as the percentage of participants who completed the module or program and agreed or strongly agreed with the indicator statement. Some indicators will be asked both in reference to specific modules and in reference to the program as a whole.

^b^We will also create a continuous program acceptability index score for each mother and teenager by summing across acceptability indicators (range 0-9).

### Data Collection

#### Overview

Data sources for assessing feasibility and acceptability outcomes described further in the following sections include (1) web analytics (eg, participant log-ins, page views, and visit times), which capture data needed to assess program adherence; (2) module completion surveys, which are embedded in the web-based program and assess program acceptability; (3) program completion surveys, administered through REDCap, which assess program acceptability and barriers to completion among all participants enrolled in the study; (4) baseline and 90-day follow-up surveys, also administered through REDCap, which access constructs in the program conceptual model (Figure 2); and (5) the web-based screener and study participant contact tracking database, which will be populated by program staff and will capture data on recruitment, eligibility, enrollment, and retention.

#### Website Use Data

Website use data will include the number of program log-ins, pages viewed, visit times, and modules completed. In addition, the participants will be offered the opportunity to rate the selected videos and activities from 1 to 5 stars. Altogether, ratings and website use data will inform our understanding of the acceptability of videos and activities and allow for the assessment of program engagement and adherence.

#### Module Completion Surveys

Brief (1- to 2-minute) module completion surveys will be embedded in the web-based program. These surveys will appear at the end of each module and solicit mother and teenager feedback on module acceptability and any technical problems encountered. Mothers and teenagers will be asked to complete separate surveys before proceeding to the next module.

#### Program Completion Surveys

Both mothers and teenagers will be asked to complete the program completion surveys, which will be administered through REDCap. The links to the surveys will be emailed to the mothers after completion of the final module. Surveys will assess program acceptability indicators (Textbox 2), as well as barriers to completion and suggestions for program improvement. Mothers and teenagers who do not complete the program within the 8-week implementation period, including those who never log into the program or those who log in but do not complete any modules, will be sent a program completion survey tailored to their completion level.

#### Baseline and Follow-up Surveys

Baseline and follow-up surveys, administered to mothers and teenagers through REDCap at enrollment and 90 days following enrollment, will assess the demographic characteristics of participants, including both the mother’s and teenager’s past exposure to IPV (baseline only), as well as cognitions, skills, and behaviors targeted by the eMTSD program. The collection of these data will allow us to describe study participants; gauge survey length and completion rates; assess psychometric properties of key measures; and explore pre-post changes in the cognition, skills, and behaviors targeted by the program (Figure 2, program conceptual model).

#### Web-Based Screener and Recruitment Tracking Data

The web-based screener will capture data on recruitment sources and participant demographics (eg, race, ethnicity, and sex). A study tracking database will capture data on staff contacts with potential participants, telephone eligibility screens, participant enrollment, program log-in, survey completion reminders, and incentive disbursements. These data will be used to explore the effectiveness of different recruitment strategies for identifying individuals who meet the study inclusion criteria by recruitment source and participant characteristics.

### Statistical Analysis

#### Overview

Descriptive statistics will be used to summarize the demographic characteristics of the study population and feasibility and acceptability indicators for the sample. Bivariate statistical procedures (eg, 2-tailed *t* tests and chi-square tests) will be used to examine associations between demographic and background characteristics of mother and teenager participants (age of the mother; sex, gender identity, and race and ethnicity of the teenager; family financial stress; mother and teenager psychological distress; and mother and teenager IPV exposure) and (1) recruitment source (eg, Facebook advertisement, domestic violence agency), (2) program adherence, (3) program acceptability, and (4) study retention. These analyses will help identify potential sources of bias and characterize program feasibility and acceptability as a function of participants’ demographic characteristics.

Descriptive statistics will be used to summarize adherence rates by *adherence support* group, and bivariate statistics will be used to examine differences between groups in adherence indicators (program log-in and initiation rates and program adherence). Paired *t* tests and McNemar test will be used to examine trends in pre-post changes in cognition, skills, and behaviors targeted for change by the program (Figure 2) among program completers. These analyses are considered exploratory, given that the study is not powered to assess changes in these constructs.

#### Power

We used the approach described by Lewis et al [29] to determine the sample size for this feasibility and acceptability trial. This approach uses a *traffic light* system to evaluate the progression to the main trial based on a set of a priori criteria. Hypothesis testing for binary feasibility outcomes tests against being in the red zone (unacceptable outcome) based on the expectation of being in the green zone (acceptable outcome). Using this approach, an adequate sample size is that which gives high power (≥80%) to reject being in the *red zone* if the *green zone* holds true.

Three focal feasibility and acceptability outcomes were selected to determine the sample size: (1) enrollment rate, (2) overall program acceptability, (3) teenager program initiations, and (4) program completion rates. The point estimates that will be considered the red zone upper limit (R_ul_) and green zone lower limit (G_ll_) for these outcomes are (1) the proportion of those eligible from those who enroll (R_ul_=0.60 and G_ll_=0.75), (2) the proportion of those who agree or strongly agree that the program is acceptable across all indicators >0.60 (R_ul_=0.55 and G_ll_=0.75), (3) the proportion of those enrolled who initiated the use of the program with their teenager >0.50 (R_ul_=0.50 and G_ll_=0.65), and (4) the proportion of those enrolled who completed the program >0.30 (R_ul_=0.30 and G_ll_=0.50). The null hypothesis test, for which the sample size is calculated, is that the true outcome is not greater than R_ul._

Table 3 shows that a sample size of 100 provides >80% power to reject the null hypothesis across each of the focal feasibility and acceptability outcomes, with an α of .05 and a 1-tailed, 1-sample binomial test based on normal approximation (with continuity correction).

**Table 3:** Power for focal binary feasibility and acceptability outcomes.

Outcome	Red zone upper limit, %	Green zone lower limit, %	Power^a^, %	N^b^
Enrollment rate (percentage of those eligible who enroll)	60	75	80	68
Program acceptability (percentage of those completing the program who agree or strongly that the program is acceptable across all domains)	55	75	80	40
Teenager program initiation rate (percentage of those enrolled who start the program with their teenager)	50	65	80	73
Program completion rate (within group; percentage of those assigned to the group who complete all 6 program modules)	25	50	80	25 (per group; 100 overall)

^a^Power (1-B) to reject being in the *red zone* if the *green zone* holds true.

^b^Needed sample size for the hypothesis test.

### Incentives

Mother and teenager participants will each receive US $30 for completing the baseline, program completion, and follow-up surveys to a total of up to US $180 per dyad for completing all study activities.

### Ethics Approval

This study was approved by the University of North Carolina at Chapel Hill Institutional Review Board on November 17, 2021 (reference number: 21-2380). Mothers who screen as eligible will be provided with information about the study and will provide electronic consent for their and their teenager’s participation. Teenagers will also complete the electronic assent form. Surveys will include referral information for support in addressing mental health and violence prevention and treatment for all participants. All project data will be deidentified before analysis and securely stored in encrypted files on servers that adhere to the University of North Carolina at Chapel Hill policy on the storage and transmission of sensitive data.

## Results

This feasibility and acceptability study began recruitment in November 2021, and the study results will be available in 2023.

## Discussion

### Anticipated Findings

We anticipate that this feasibility and acceptability study will determine whether the eMTSD program merits rigorous testing in future hybrid effectiveness-implementation trials. Such a trial may be designed to examine program effects on ADV outcomes and the impacts of delivery mode (web-based vs booklet) and implementation support (eg, reminders and action planning) on D&I outcomes. Furthermore, we expect that the results of this study will inform our understanding of optimal recruitment strategies and identify potential changes to the delivery and content of the program and implementation support that would improve acceptability and feasibility.

### Comparison With Prior Work

MTSD is one of the few violence prevention programs that has been developed and tested with youth exposed to IPV, and to the best of our knowledge, is the only program that targets risk and protective factors at both the family and individual levels [8]. The feasibility and acceptability study of the web-based version of this program will contribute to a small but growing body of research examining the use of technology and digital delivery methods for prevention programs that aim to make changes to the family environment and, in turn, prevent negative health outcomes among youth [26,30-32].

### Strengths and Limitations

The limitations of this study include the use of a convenience sample, which may preclude our ability to generalize findings to the population as a whole and explore differences in findings across subgroups (eg, youth of different gender identities and youth who have been exposed to different types of caregiver IPV). In addition, survey data rely on self-reporting and are, thus, potentially susceptible to social desirability response bias. The strengths of the study include the use of a multipronged recruitment strategy, which will allow us to explore the effectiveness of different recruitment sources and the testing of different implementation strategies (action planning and text reminders) that may increase program adherence.

### Conclusions

If eMTSD is acceptable and effective in preventing ADV, it is hoped that the program may be promoted by organizations serving survivors of IPV and their families. Given the dearth of programs designed specifically for youth exposed to IPV, eMTSD may address a critical gap in ADV prevention efforts and decrease the likelihood of abusive dating and intimate partnerships during adolescence and adulthood, as well as the adverse health and social impacts of ADV and IPV.

## Appendix

Multimedia Appendix 1 Program activity screenshots.

## Abbreviations

ADV: adolescent dating violence
D&I: dissemination and implementation
eMTSD: eMoms and Teens for Safe Dates
IPV: intimate partner violence
MTSD: Moms and Teens for Safe Dates
REDCap: Research Electronic Data Capture
TFA: Theoretical Framework of Acceptability
