# Supplementary material for: Web-Based Delivery of a Family-Based Dating Violence Prevention Program for Youth Who Have Been Exposed to Intimate Partner Violence: Protocol for an Acceptability and Feasibility Study
Source: JMIR Res Protoc. 2022 Aug 5;11(8):e35487. doi: 10.2196/35487 (PMC9391968; doi:10.2196/35487)

## Appendix 1. Screen Shots of eMTSD Program Activities

### Getting Started Module Activity


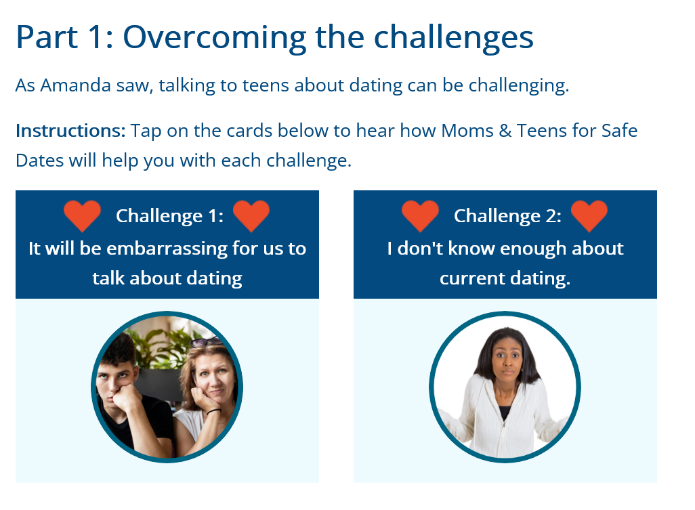


### Home Page


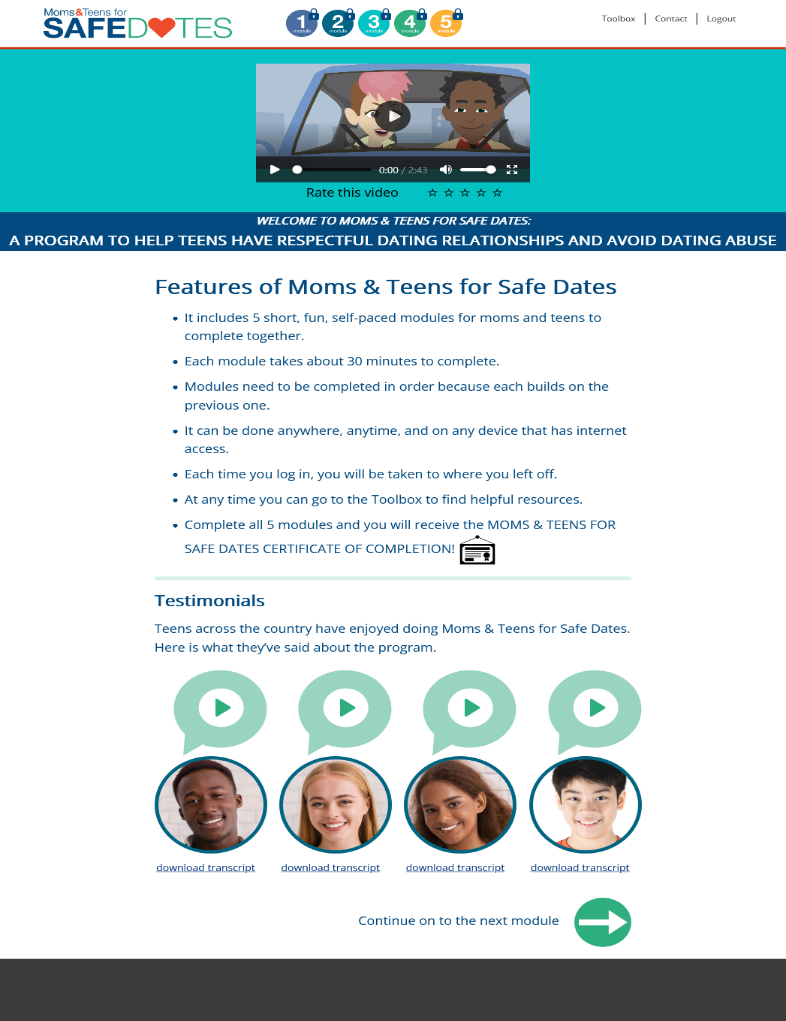


### Module 1 Activity


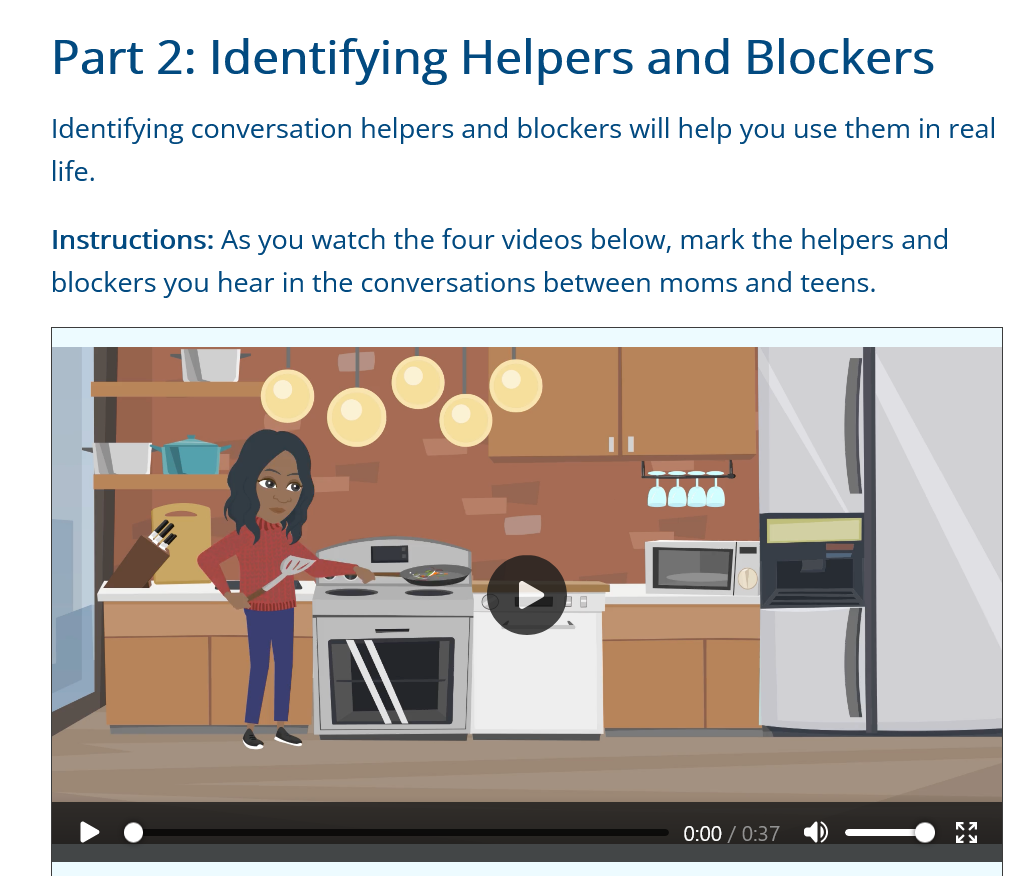


### Module 2 Activity


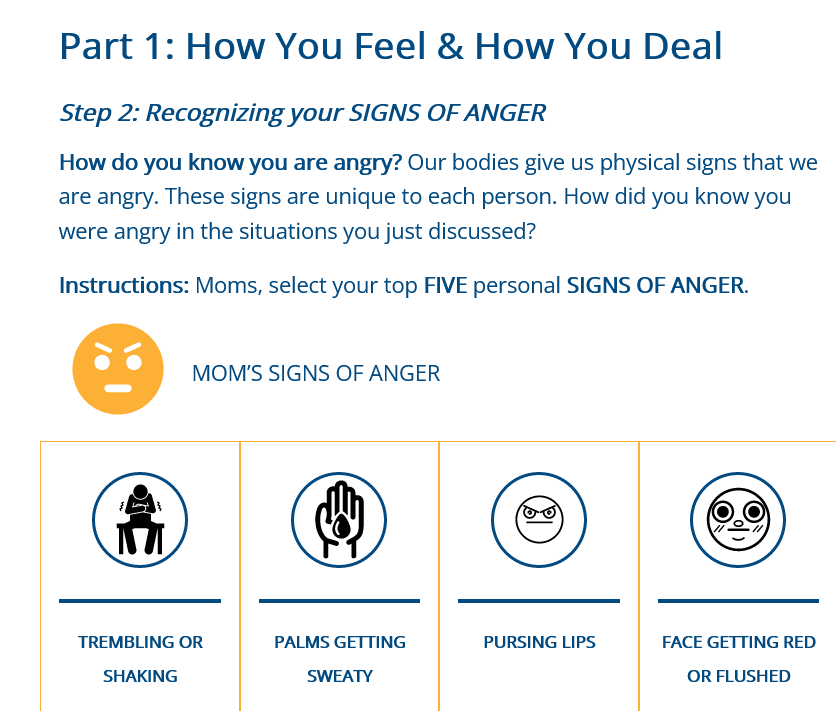


Module 3 Activity


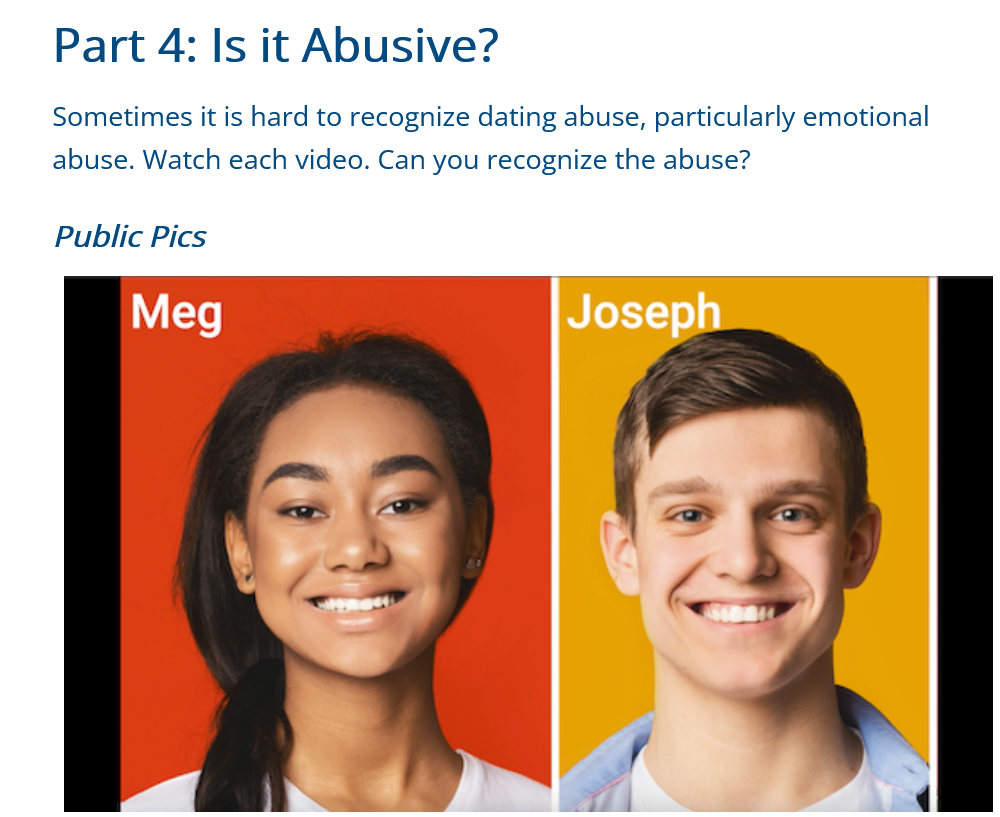


Module 4 Activity


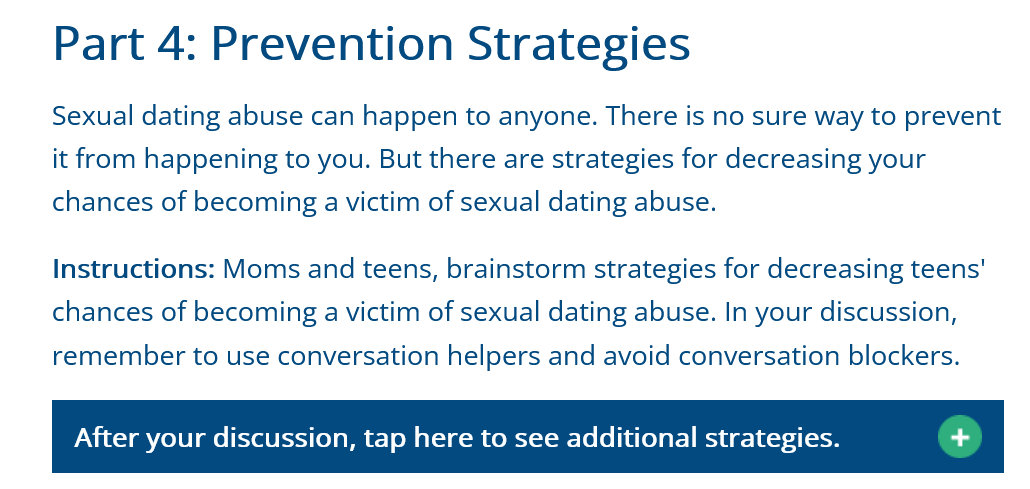


### Module 5 Activity


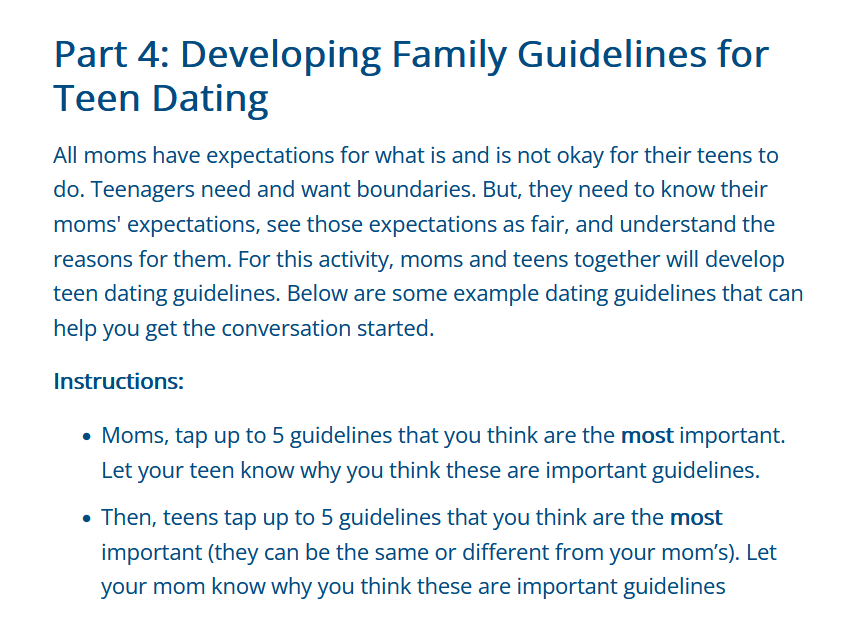

Supplement: Multimedia Appendix 1 [file resprot_v11i8e35487_app1.docx]
